# Differential fates of introns in gene expression due to global alternative splicing

**DOI:** 10.1007/s00439-021-02409-6

**Published:** 2021-12-14

**Authors:** Anjani Kumari, Saam Sedehizadeh, John David Brook, Piotr Kozlowski, Marzena Wojciechowska

**Affiliations:** 1 Queen’s Medical Centre, School of Life Sciences, University of Nottingham, Nottingham, NG7 2UH UK; 2 Department of Molecular Genetics, Institute of Bioorganic Chemistry, Polish Academy of Sciences, 61-704 Poznan, Poland; 3 Present Address: Department of Rare Human Diseases, Institute of Bioorganic Chemistry, Polish Academy of Sciences, 61-704 Poznan, Poland

## Abstract

The discovery of introns over four decades ago revealed a new vision of genes and their interrupted arrangement. Throughout the years, it has appeared that introns play essential roles in the regulation of gene expression. Unique processing of excised introns through the formation of lariats suggests a widespread role for these molecules in the structure and function of cells. In addition to rapid destruction, these lariats may linger on in the nucleus or may even be exported to the cytoplasm, where they remain stable circular RNAs (circRNAs). Alternative splicing (AS) is a source of diversity in mature transcripts harboring retained introns (RI-mRNAs). Such RNAs may contain one or more entire retained intron(s) (RIs), but they may also have intron fragments resulting from sequential excision of smaller subfragments via recursive splicing (RS), which is characteristic of long introns. There are many potential fates of RI-mRNAs, including their downregulation via nuclear and cytoplasmic surveillance systems and the generation of new protein isoforms with potentially different functions. Various reports have linked the presence of such unprocessed transcripts in mammals to important roles in normal development and in disease-related conditions. In certain human neurological-neuromuscular disorders, including myotonic dystrophy type 2 (DM2), frontotemporal dementia/amyotrophic lateral sclerosis (FTD/ALS) and Duchenne muscular dystrophy (DMD), peculiar processing of long introns has been identified and is associated with their pathogenic effects. In this review, we discuss different mechanisms involved in the processing of introns during AS and the functions of these large sections of the genome in our biology.

## Introduction

Introns, as well as their splicing and retention were discovered in 1977 as a result of an interesting observation that the mRNA used to code for proteins was almost always shorter than the DNA from which it had been transcribed (Berget and Sharp 1977; Chow et al. 1977). The original publications described the discovery in adenovirus mRNAs, but it soon became clear that this mechanism was not solely a viral phenomenon since introns were also found in cellular genes from eukaryotic cells (Breathnach et al. 1977; Jeffreys and Flavell 1977; Mandel et al. 1978)*.* Initially, genes that contained introns were called interrupted and considered to be noncoding; however, this notion has changed over time. The completed sequencing projects of the human genome and other organisms including *D. melanogaster* and *C. elegans* have confirmed that all eukaryotes have introns (Rogozin et al. 2012). However, different species harbor dramatically different density and length of introns, ranging from a few bps to hundreds kbps. Genes in higher eukaryotes such as mammals have a greater number of introns than those of lower eukaryotes such as yeast, *Drosophila*, and *C. elegans* (Nixon et al. 2002; Wu et al. 2013). The differences may partly be explained by the variability in modes of intron removal between these organisms. Several studies revealed that first introns (i.e., the 5ʹ most first introns of genes) are typically the longest and most conserved (Gaffney and Keightley 2004; Bieberstein et al. 2012). Conservation of the first intron is probably related to the presence of regulatory elements and a specific pattern of chromatin organization. In the human genome, exceptionally long introns are found in genes with a wide variety of cellular and developmental functions, including genes with important roles in some diseases, e.g., *dystrophin* in DMD and c*ystic fibrosis transmembrane conductance regulator* (*CFTR*) in cystic fibrosis (CF) (Sterrantino et al. 2021). The number of introns in transcribed human RNAs ranges from zero in histones to over 75 introns in *dystrophin*, in which only 0.5% of the gene is comprised of exons. Similarly, *CFTR* is approximately 98% introns (Bonadia et al. 2014). Such vast stretches of the genome must have an important biological function, given the amount of energy expended by the cell in the synthesis and transcription of these seemingly useless pieces of DNA. The fact that all higher metazoan species have introns, and that the higher the organism is, the higher the proportion of introns, indicates their important biological role.

**Fig. 1:**
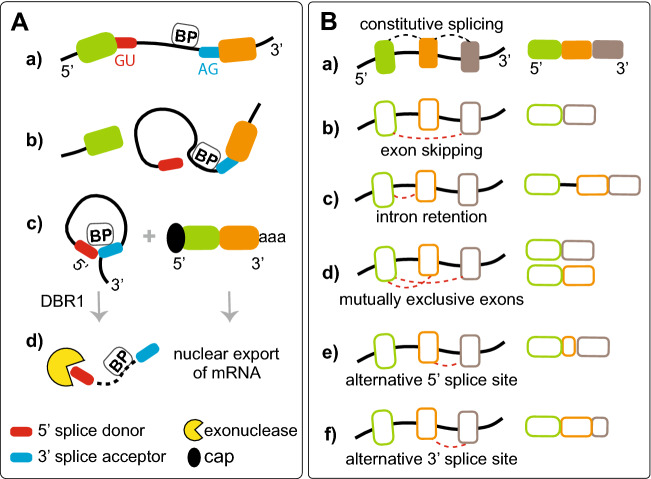
Constitutive and alternative pre-mRNA splicing. **A** *Processing of introns during constitutive splicing.* **a** Intron excision from pre-mRNA requires the presence of specific dinucleotide sequences located at its termini, i.e., a splice donor (conserved GU at the 5′ss) and a splice acceptor (conserved AG at the 3′ss), in addition to the branch point (BP) site. **b** The first step involves cleavage of the pre-RNA at the 5′ss of the intron followed by the joining of the 5′ end to an adenosine unit (**A**) embedded within a BP sequence via a 2ʹ, 5ʹ-phosphodiester bond. **c** Release of the intron as the lariat is formed by the ligation of the 5′ and 3′ splice sites of exposed exons. **d** Excised lariat RNA is subsequently debranched by the DBR1 enzyme, which leads to its linearization followed by rapid degradation in the nuclear turnover pathway, while mature mRNA is exported to the cytoplasm. **B** *Types of alternative splicing events*. **a** Schematic representation of constitutive splicing and (**b**–**f**) alternative splicing events. The exons are depicted by colored empty and filled boxes and introns are shown as black solid lines. Dashed black and red lines indicate constitutive and alternative splicing events, respectively

Removal of introns through editing of premature RNA (pre-mRNA) requires the presence of splice donor (5′ss) and splice acceptor (3′ss) sites, in addition to the branch point (BP) site located near the 3′ss. This process involves the formation of a lariat intermediate controlled by specific dinucleotide sequences that border the exon/intron junction, i.e., conserved GU and AG, respectively, at the 5′- and 3′-ends of each intron (Fig. 1A). In human genes, the BP site is a short motif comprised mostly of adenosine, and the sequence is located between 18 and 44 nt upstream of the 3’ss; however, some BPs can be more distant and are found up to 400 nt upstream of the 3’ end (Gooding et al. 2006; Gao et al. 2008; Mercer et al. 2015). Editing of the pre-mRNAs to release introns and produce a functional unit is confined within the nucleus. This process is mediated by the spliceosome, a large ribonucleoprotein (snRNP) complex composed of small nuclear RNAs (snRNAs) and a few dozen proteins. The first step involves cleavage of the precursor RNA (pre-RNA) at the 5′ss of the intron followed by the joining of the 5′ end to an adenosine unit embedded within a BP sequence via a 2ʹ,5ʹ-phosphodiester bond, and eventually, the intron undergoing processing only remains attached to the downstream exon. Its release in the form of the lariat results from the ligation of the 5′ and 3′ splice sites of exposed exons and the ligation process is facilitated by multiprotein Exon Junction Complex (EJC) that is deposited ~ 20–24 nucleotides upstream of exon-exon junctions during mRNA splicing (Joseph and Lai 2021). Released lariat RNA is subsequently debranched by the RNA lariat debranching (DBR1) enzyme. This leads to its linearization and rapid degradation by the intron turnover pathway (Fig. 1A). A variety of exceptions to this canonical pathway have been described, and experimental evidence has shown that some lariat RNAs may escape the debranching process. These escapees either persist longer in the nucleus or travel to the cytoplasm, where they remain as stable circular RNAs (Talhouarne and Gall 2014, 2018).

Transcriptome diversity in eukaryotes may occur through alternative splicing (AS) of pre-RNAs, through the use of alternative promoters and polyA signals and, to some extent, through nucleotide editing. Intron retention (IR), which was originally described in viruses is one type of alternative splicing event (ASE) and is characterized by the inclusion of one or more introns in mature mRNA (RI-mRNA). This class of ASE is distinct from other types in that the mature transcript harboring unspliced introns still contains a potentially spliceable unit (Sznajder et al. 2018; Monteuuis et al. 2019; Broseus and Ritchie 2020). Although IR events have been found to be an integral part of global alternative splicing in mammals, the mechanism and factors underlying IR remain poorly understood (Wong et al. 2013, 2016, 2017; Braunschweig et al. 2014; Pimentel et al. 2016; Edwards et al. 2016). It was proposed that IR controls mammalian gene expression via a global, bidirectional cross-talk mechanism, in which inaccurate transcription results in low levels of expression due to impaired recruitment of core splicing factors (Wong et al. 2017; Zhang et al. 2018). This in turn results in localized pausing of RNA Pol II, further intron retention, and ultimately turnover of the RI-mRNAs (Zhang et al. 2018). Intron retention regulates RNA stability and protein isoform production in various ways. When in the main open reading frame (ORF), IR may either maintain the frame to allow translation of IR transcripts, or it may interrupt the ORF by introducing a premature termination codon (PTC). Due to the frameshift, these translation-incompetent mRNAs are eventually turned over via the cytoplasmic surveillance machinery of nonsense-mediated decay (NMD) (Lewis et al. 2003; Weischenfeldt et al. 2012; Ge and Porse 2014). However, some RI-mRNAs with PTCs are translated into new protein isoforms. This phenomenon was first discovered in viruses which developed two pathways for nuclear export of mRNAs with retained introns that allow them to escape either the nuclear or cytoplasmic degradation machineries and subsequently permit such incompletely spliced mRNAs to be translated (Hadzopoulou-Cladaras et al. 1989; Grüter et al. 1998; Hammarskjöld et al. 1989). These mechanisms rely on the interactions of *cis*-acting elements in RI-mRNAs with nuclear export proteins. Although they were originally thought to be unique to viruses, it was subsequently shown that the mammalian nuclear export factor 1 (*NXF1*) gene itself contains a constitutive *cis*-acting transport element in its alternatively spliced mRNA with a retained intron (Li et al. 2006). Direct interaction of Nxf1 with this element enables export and translation of a short alternative Nxf1 protein (Li et al. 2016a, b). Additionally, IR may be a source of mRNA isoforms referred to as detained introns (DI), representing either nuclear end-products or stable intermediates, which are temporarily stored awaiting signals for splicing completion and nuclear export (Ninomiya et al. 2011; Boutz et al. 2015; Mauger et al. 2016). Nuclear retention of introns has also been implicated in the pathogenesis of human hereditary disorders, including myotonic dystrophy type 2 (DM2, *CNBP* locus with CCTG^exp^), Fuchs endothelial corneal dystrophy (FECD, *TCF4* locus with CTG^exp^), and amyotrophic lateral sclerosis/ frontotemporal dementia (C9-ALS/FTD, *C9orf72* locus with GGGGCC^exp^) (Sznajder et al. 2018). Peculiar processing of introns harboring these GC-rich microsatellites has been linked with their propensity to adopt secondary RNA structures which may limit recruitment of splicing machinery factors and eventually may perturb proper removal of the introns from their host transcripts (Mirkin 2007; Zhang and Ashizawa 2017).

During the canonical splicing of pre-RNA, an intron is removed as a single unit in a two-step reaction. However, some large introns (> 10 k nt) pose challenges during processing and are cut out by splicing of distinct segments in a process known as recursive splicing (RS) (Joseph et al. 2018). This multistep intraintronic splicing was first discovered in *Drosophila* and was later also observed in genes of the human transcriptome (Hatton et al. 1998; Burnette et al. 2005). Despite the fact that approximately half of human protein-coding genes have introns over 24 k nt long and contain motifs similar to *Drosophila* RS-sites, only a handful of recursively spliced introns was initially identified in humans, mostly in genes involved in brain development, despite the greater abundance of long introns in vertebrate genomes (Duff et al. 2015; Sibley et al. 2015). However, recent study has revealed that most introns of human genes are removed from pre-mRNAs in smaller pieces rather than spliced as whole units in one step reaction (Wan et al. 2021). These results have led to a model of stochastic splice site selection based on which unannotated splicing sites (internal RS sites) within introns are used frequently but randomly by the spliceosomes to make many cuts instead of a single cut to progressively remove an intron. This process results in the generation of transient splicing intermediates that are pools of final mRNAs. Little is known about the RS machinery involved in the processing of RS introns; however, it was proposed that it is kinetically coupled with RNA polymerase II (RNA Pol II) elongation. In mammalian cells, most RS events occur posttranscriptionally, and RS introns are removed by both recursive and canonical splicing. The choice of which splicing mechanism is used seems to be cell-type specific (Zhang et al. 2018).

In this review, we present various pathways of intron processing during alternative splicing of mammalian cells and tissues. We discuss the functions and fate of mature mRNAs harboring unspliced introns in physiological and pathological programs of global alternative splicing that mediate gene expression. Finally, we discuss the most recent data on stable circular intronic RNA in various vertebrates, for which biogenesis, biological functions and turnover remain to be investigated.

## Aberrant processing of introns during global alternative splicing

The formation of fully functional mRNA involves the processing of a precursor transcript through three main modifications: 5′-capping, 3′-polyadenylation and splicing. The diversity of the transcriptome in eukaryotes results from alternative splicing of pre-RNAs. High-throughput transcriptome analyses have shown that AS affects approximately 95% of multiexonic genes in humans (Nilsen and Graveley 2010; Barbosa-Morais et al. 2012; Merkin et al. 2012). During AS, splice sites in primary transcripts are differentially utilized, making it possible for a single gene to produce multiple mRNA and protein isoforms. Through this mechanism of regulating gene expression, the information stored in the genes can be processed in a variety of ways (Brett et al. 2002; Roy et al. 2013). Alternative splicing is regulated by the complex interplay between *cis*- and *trans-*acting factors that serve to promote or repress the assembly of splicing complexes, referred to as spliceosomes. Tight control of AS has paramount importance, as evidenced by links between abnormal alternative splicing and human disorders, and over the past years, ASEs have been recognized as promising biomarkers for clinical diagnosis and targets for therapeutic intervention (Cooper et al. 2009; Dvinge and Bradley 2015; Jung et al. 2015). ASEs can be classified into different splicing patterns that include alternative cassette exons (i.e., exon inclusion or exclusion), mutually exclusive exons, retained introns and exitrons, alternative 5′ and 3′ splice sites, alternative promoters, and alternative poly-A sites (Fig. 1B). Exon inclusion and exclusion have long been recognized as the most frequent types of AS in animals and implicated in the control of diverse aspects of normal and disease biology. However, the development of RNA-Seq over the past decade has highlighted the importance of recognizing intron retention (Kalsotra and Cooper 2011; Braunschweig et al. 2014; Wong et al. 2016; Pimentel et al. 2016). Although IR events have been found to be an integral part of many mammalian physiological and pathological programs of the global alternative splicing-mediated regulation of gene expression, this type of ASE remains much better characterized in plants, fungi, and unicellular eukaryotes. In these organisms, IR is the major mechanism for the regulation of gene expression (Ner-Gaon et al. 2004; Syed et al. 2012).

**Fig. 2:**
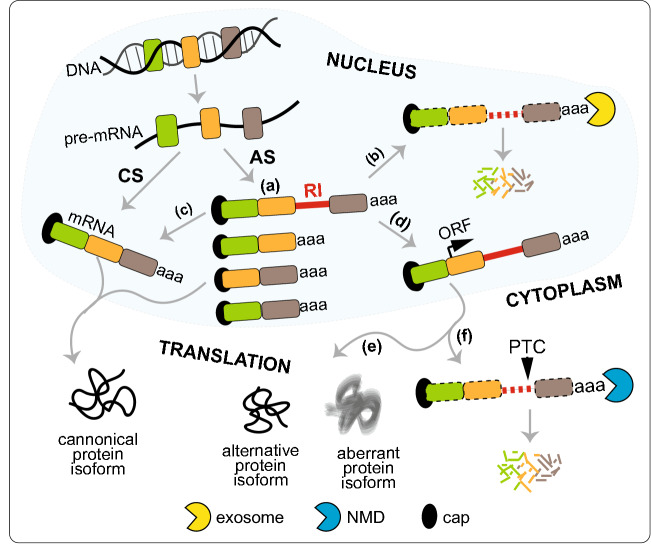
The fate of transcripts harboring retained introns (RI-mRNAs). **a** Alternative splicing may generate mature transcripts with one or more retained introns (RIs). The outcomes of retention may be different. **b** The RI-mRNA may represent a nuclear end-product guided to exosome-mediated RNA turnover of the host transcripts. **c** It may also represent a nuclear stable intermediate containing a potentially spliceable intron. In this scenario, the RI-mRNA is temporarily stored in the nucleus awaiting signals for splicing completion and export. Both (**b**) and (**c**) represent examples of transcripts with detained introns. **d** RI in the main ORF can either maintain the frame to allow translation of RI-mRNA to generate alternative protein isoforms with novel functions or aberrant protein isoforms (**e**), or it may interrupt the ORF of the mRNA, leading to introduction of PTC and resulting in the generation of translation-incompetent mRNAs due to frameshift (**f**). The detection and ultimate degradation of such transcripts is maintained via the cytoplasmic surveillance machinery via NMD (**d**). CS, conventional splicing; AS, alternative splicing; aaa, poly(A) tail; filled colored boxes, exons

Incompletely spliced transcripts may harbor one or more the entire introns but may also contain intron fragments resulting from piecewise processing of large introns of > 10 k nt via recursive splicing. Initially, such RI-mRNAs were considered to be nonproductive based on the RNA isoform being predestined for degradation rather than translated. However, in many cases, these products do not lack functionality and play important regulatory roles (Middleton et al. 2017; Jacob and Smith 2017; Schmitz et al. 2017). The fate of IR-harboring transcripts depends upon a number of factors, including the location of the IR event within the transcript. IR in the 3′-UTR can introduce *cis*-elements, affecting mRNA stability or translational efficiency; IR in the 5′-UTR can insert an upstream ORF (uORF) or other structural features that can activate or repress translational initiation efficiency (Tahmasebi et al. 2016). More commonly, intron retention that interrupts the main ORF may lead to the introduction of PTC. The detection and ultimate degradation of such transcripts is maintained through the cytoplasmic surveillance machinery, which downregulates gene expression via NMD (Lewis et al. 2003; McGlincy and Smith 2008; Weischenfeldt et al. 2012; Ge and Porse 2014; Lareau and Brenner 2015). Importantly, some RI-mRNAs with PTCs are translated into new protein isoforms (for more details, please see the section Intron Retention in Viruses). However, IR in the main ORF can also maintain the reading frame, allowing translation of IR transcripts to generate alternative protein isoforms with novel functions. IR may also generate mRNA isoforms referred to as detained introns representing the nuclear end-products and nuclear stable intermediates (Fig. 2). In general, the first group is allocated to exosome-mediated RNA turnover of the host transcripts, whereas the second group of RNA containing a potentially spliceable intron is temporarily stored, awaiting signals for splicing completion and is eventually exported. Transcripts with such introns are considered a reservoir for rapid induction of expression without the necessity for transcription initiation (Boutz et al. 2015; Mauger et al. 2016). In addition to the above IR events, an unusual subfamily of introns has been discovered inside annotated protein-coding exons of plant and human genomes (Marquez et al. 2012, 2015). Based on their exonic and intronic nature, they were termed exitrons (exonic introns, EIs). These EIs have all canonical core splicing signals (5′ and 3′ splice sites and branch points), but they do not contain stop codons. Although EIs were detected as a subgroup of retained introns, they are clearly distinguishable. Human and Arabidopsis exitrons share some features, i.e., (i) weaker splice sites and higher GC content in comparison to RIs; (ii) size that is closer to exons than to RIs; and (iii) EI-containing exons are considerably longer than other exons (Marquez et al. 2015). EI removal or retention through alternative splicing results in transcripts with different fates. Since exitrons are protein-coding sequences, they have the potential to increase proteome complexity and add to phenotypic diversity via AS. In plant and nonplant species, EI-containing isoforms are exported to the cytoplasm, associate with ribosomes and are translated in contrast to IR transcripts that are often retained in the nucleus and are not translated (Yap et al. 2012; Shalgi et al. 2014).

A number of research groups have used various approaches for monitoring the extent to which RNA isoforms of AS associate with ribosomes and whether the isoforms are differentially translated (Sterne-Weiler et al. 2013; Shalgi et al. 2014). These studies found that IR events were detectable in cytoplasmic fractions and engaged with ribosomes to variable extents. High-resolution fractionation of polysomes into different size classes revealed that IR events were mostly present in a cluster of poorly translated transcripts, and only a small number of IR-containing transcripts were enriched in larger polysomes (Floor and Doudna 2016). As an alternative to polysome profiling, Weatheritt et al. combined ribosome footprinting with RNA-Seq to assess ribosomal engagement by mRNA isoforms (Weatheritt et al. 2016). This study revealed that IR was underrepresented on ribosomes compared to whole-cell RNA. In addition, IR events were enriched among the lowest expressed RNAs, consistent with ribosome engagement as a precursor to NMD. Thus, there are various ways in which intron retention regulates RNA stability and protein isoform production, as well as a rapid induction of expression via posttranscriptional splicing of nuclear-detained introns.

## Intron retention in viruses

Intron retention was initially recognized in the analysis of viral interactions with host cells, and a mechanism for export and expression of mRNA with retained introns was first studied in human immunodeficiency virus (HIV) (Hadzopoulou-Cladaras et al. 1989; Hammarskjöld et al. 1989). In the late 1980's it was observed that HIV and other retroviruses use one of two mechanisms to overcome cellular restrictions to export and translation of their unspliced and incompletely spliced mRNAs. In complex retroviruses, such as HIV, virus-encoded regulatory proteins (Rev for HIV) bind to a specific *cis*-acting element in the incompletely spliced viral mRNA (RRE for HIV) and contact a host cell karyopherin protein (CRM1), also known as Exportin1 or Xpo1. Subsequently, this complex shuttles to the cytoplasm, where it delivers the RNA cargo (Hadzopoulou-Cladaras et al. 1989; Malim et al. 1989; Hammarskjöld et al. 1989; Fornerod et al. 1997). Thus, Rev works in *trans* on the RRE, and if this element is mutated or deleted or if Rev is not present, viral mRNAs with retained introns are not exported from the nucleus of the host cell. The specific domain in Rev that recruits Crm1 was identified to have a “nuclear export signal” (NES) (Fischer et al. 1995). Similar to many cellular proteins with the NES, Rev also has a nuclear localization signal (NLS) and shuttles between the nucleus and the cytoplasm even when it is not bound to RRE-RNA (Meyer and Malim 1994; Kalland et al. 1994). On the other hand, simpler retroviruses also contain a *cis*-acting sequence in their mRNAs, a constitutive RNA transport element (CTE) (Bray et al. 1994; Rekosh and Hammarskjold 2018). This element functions constitutively in host cells without a viral protein and interacts directly with the NXF1 to enable nuclear exit and subsequent translation of RI-mRNAs (Grüter et al. 1998). Although these mechanisms were originally thought to be unique to viruses, it was subsequently shown that the mammalian *NXF1* gene itself contains a CTE in an alternatively spliced mRNA with a retained intron (Li et al. 2006). Direct interaction of Nxf1 with the CTE enables export and translation of a short alternative Nxf1 protein. It is still unclear how many mammalian genes contain elements that can function as CTEs. However, there are now numerous examples of RI-mRNAs that are efficiently exported from the nucleus and used to express novel proteins (Li et al. 2006; Yap et al. 2012; Pimentel et al. 2016).

## *Cis-* and *trans*-acting elements regulate intron retention

**Fig. 3:**
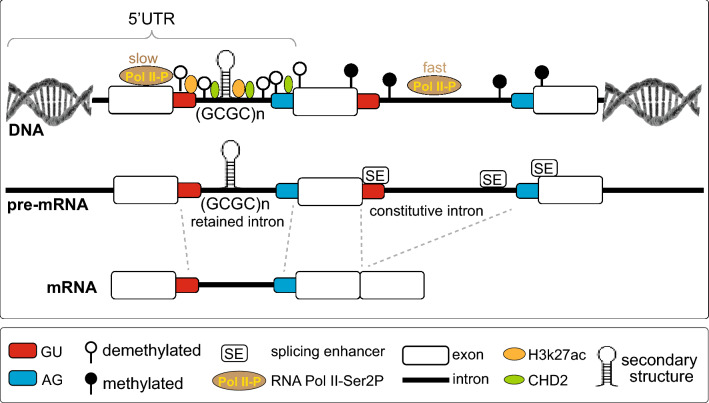
Regulation of intron retention by cis-and trans-acting elements. IR is dependent on the location of the intron within the gene and is enriched in UTRs. Among distinct *cis* features that discriminate the retained introns from general introns undergoing constitutive splicing are reduced length, elevated GC content, demethylated CpG, and weaker 5′ and 3′ splice sites. Intron retention is also marked by an enrichment of *trans-*acting factors, such as chromodomain-helicase-DNA-binding protein 2 (CHD2), and specific modification of chromatin, i.e., acetylation of histone H3 (H3K27ac). Additionally, splice junctions of the retained introns are sites for pausing the phosphorylated form of RNA Pol II at serine 2 (Pol II-Ser2P) associated with transcription elongation. This coincides with inefficient recruitment of *trans*-acting spliceosomal components. Importantly, the adaptation of secondary structures formed by some repetitive sequences of DNA and RNA within introns may have an adverse effect on their splicing

The widely known factors of splice signal recognition that are integral to AS include epigenetic changes (e.g., DNA methylation), RNA Pol II processing, and splicing factor recruitment (Shukla et al. 2011). Their relevance to control IR has been a matter of investigation and has resulted in the recognition of both *cis*- and *trans-*acting elements as contributors to the mechanisms underlying IR-mediated regulation of gene expression (Braunschweig et al. 2014; Wong et al. 2017). Analysis of poly(A)^+^ RNA-Seq data from diverse mammalian and murine tissues and cell types revealed some distinct features that discriminate the retained introns from general introns undergoing constitutive splicing. They include elevated GC content, increased demethylated CpG, reduced length and weaker 5′ and 3′ splice sites (Fig. 3). Additionally, IR was found to be dependent on the location of the intron within the gene and was enriched in UTRs, but depleted in protein-coding regions. These features were characteristic of transcripts either from nuclear or cytoplasmic mammalian poly(A)^+^ RNA. In the search for *cis*-regulatory elements and *trans*-acting factors, Braunschweig and colleagues analyzed ENCODE ChIP-Seq data from human (K562 hematopoietic) and mouse (CH12 B lymphoma) cell lines focusing on transcription and chromatin components. They detected significant enrichment of RNA Pol II and some of the chromatin regulators, e.g., chromodomain-helicase-DNA-binding protein 2 (CHD2), over retained introns compared to constitutive, and specific chromatin modifications, i.e. acetylation of histone H3 (H3K27ac). Of all the factors analyzed, the strongest enrichment was found for RNA Pol II phosphorylated at serine 2 (Pol II-Ser2P) associated with transcription elongation, indicating that RIs are sites of pausing for Pol II, whose levels appear to be tightly coupled with increased IR events (Braunschweig et al. 2014). The relationship between these two factors was confirmed after treatment of murine embryonic stem cells (ESCs) with 5,6-dichloro-1-beta-D-ribofuranosylbenzimidazole (DRB), a compound that prevents polymerase elongation by blocking phosphorylation of its C-terminal domain. Over 70% of IR events with an increased percent of IR (PIR) were detected in DRB-treated cells compared to mock-treated cells used as a control (Braunschweig et al. 2014).

DNA methylation occurs predominantly at CpG dinucleotides in mammals, and sharp transitions in its patterns are known to mark splice junctions. This suggests that these signals may be crucial for the recognition of exons and introns (Laurent et al. 2010; Jones 2012). Changes in DNA methylation levels have well-established roles in the regulation of splicing including exon inclusion and skipping (Shukla et al. 2011; Maunakea et al. 2013). To determine whether this *cis*-acting element is associated with IR, Wong and colleagues performed whole-genome bisulfite sequencing (WGBS) on genomic DNA from mouse promyelocytes and granulocytes. They measured levels of DNA methylation near the 3’ and 5’ splice junctions and within the body of all introns in these cells. Significantly reduced methylation levels were observed in retained introns than in constitutive introns, particularly near the junctions of RI; however, a weaker reduction was also detected within the body of the unspliced introns (Wong et al. 2017). Furthermore, they determined the extent of IR events that are marked by reduced DNA methylation by analyzing the methylome of other normal and cancer cell types using publicly available RNA-Seq data from humans and mice, including human ESC lines (H1 and H9), a human lung fibroblast cell line (IMR90), a human neuron progenitor cells, a human colon cancer cell line (HCT116) and mouse primary and reprogrammed fibroblasts. Similar to what was observed in murine cells, the outcomes of this study revealed that reduced DNA methylation levels consistently marked retained introns near splice junctions and within introns, indicating conservation of this phenomenon in human and mouse biology.

RNA Pol II stalling and reduced DNA methylation levels observed near splice junctions of RIs suggest that methylation regulates retention by altering RNA Pol II progression and splice site recognition. Alternatively, RNA Pol II stalling near splice junctions of retained introns could occur as a consequence of inefficient recruitment of *trans-*acting spliceosomal components (Fong and Zhou 2001; Lin et al. 2008). The latter scenario is possible since retained introns have weaker splice sites than constitutive introns and thus might not be properly recognized as being sensitive to the levels of splicing factor concentrations (Sakabe and de Souza 2007; Wong et al. 2013; Braunschweig et al. 2014). This correlation was investigated by Braunschweig and colleagues who analyzed poly(A)^+^ RNA-Seq data generated from HeLa cells following knockdown of spliceosomal components (Saltzman et al. 2008; Braunschweig et al. 2014). The authors found that (i) reduction of snRNPs resulted in an increase of PIR for retained versus constitutive introns; (ii) introns that displayed the largest increases in PIR were associated with significantly higher levels of RNA Pol II occupancy, and (iii) the levels of transcripts containing introns that show increased retention upon snRNPs depletion are significantly reduced compared to the levels of transcripts with introns that do not show an increased PIR. Based on these result, it was proposed that depletion of snRNPs contributes to increased PIR, further supporting the conclusion that IR controls mammalian gene expression via a global, bidirectional cross-talk mechanism (Braunschweig et al. 2014). In this process, inaccurate transcription and low levels of expression resulting from impaired recruitment of core splicing factors, are linked with localized pausing of RNA Pol II, retention of introns and ultimately turnover of transcripts in mammalian cells and tissues.

An interesting model of splicing control centered on the competition of pre-mRNAs for limiting the splicing apparatus was proposed by the Ares lab (Munding et al. 2013). Based on this model, the trans-competition control (TCC) model originally described in yeast, splicing regulation is subjected to the composition of a pool of endogenous competing RNAs. By altering the competitive status of a target pre-mRNA through modulation of the levels of other RNAs that compete for limiting splicing machinery availability, splicing regulation can be achieved. There are thousands of competing introns within a cell, each with its own affinity for the spliceosome; as the concentration of any one of them changes, the splicing efficiency of all the others then must change as well. Munding and coauthors demonstrated that competition between introns from expressed vegetative or meiotic genes controls the expression of genes required for sporulation in yeast. Essentially, introns from sporulation genes display weaker splice sites and are outcompeted for limited splicing factors by the much more abundant introns from ribosomal protein genes. When yeast cells are stressed, the expression of ribosomal protein genes decreases, and transcripts from meiotic genes can be spliced. The TCC model also applies to mammalian systems in which induction of gene expression programs can result in large changes in the composition of the transcript pool, altering competition for the splicing machinery (Berg et al. 2012). Under these conditions, the competitive advantage of alternative exons for the splicing machinery may be decreased, resulting in a shift of mRNA isoforms.

## Intron retention in normal biology

Alternative splicing facilitates biodiversity through the generation of various transcriptome and proteome isoforms and has been linked with developmental regulation (Wang et al. 2015). A number of studies on transcriptome-wide analyses have demonstrated that different IR events control gene expression programs during both normal development and disease-related states. For example, increased IR incidences were observed in various cells of the hematopoietic lineage, such as transformation of erythroblasts, megakaryocytes, granulocytes and CD4 T-cells (Wong et al. 2013; Pimentel et al. 2016; Edwards et al. 2016; Ni et al. 2016), during differentiation of ESCs into neural progenitors, reprogramming of mouse embryonic fibroblasts to induced pluripotent stem cells (IPSCs) (Braunschweig et al. 2014; Hussein et al. 2014; Boutz et al. 2015), and during normal development of smooth muscle cells (Llorian et al. 2016). Comparative analysis of these studies has revealed the common and unique features of IR-mediated regulation including its tissue- and cell-type specificities. In normally regulated programs involved in the developmental process, a common tendency for increased intron retention was found in more differentiated or postmitotic cellular states. However, a high percentage of IR events was not necessary for the induction of terminal differentiation. More plastic cell types whose functional maturation involves transition to proliferative states exhibit decreased IR incidence as shown during dedifferentiation of smooth muscle cells (Wong et al. 2013; Cho et al. 2014; Braunschweig et al. 2014; Pimentel et al. 2016; Edwards et al. 2016; Llorian et al. 2016). Some differentiation programs involve more complex patterns of IR for example, the final stages of erythropoiesis include a progressive increase in IR events before achieving a terminal stage that exhibited lower IR than the precursor cells. This dynamic pattern is further complicated by the presence of subgroups of IR events that exhibit differential regulation between the different stages of erythroblast differentiation (Pimentel et al. 2016; Edwards et al. 2016).

To investigate the incidence of IR, as well as its regulation and biological roles, Braunschweig et al. performed a comprehensive analysis of over forty diverse human and mouse cell and tissue types using high-coverage poly(A)^+^ RNA-Seq data (Braunschweig et al. 2014). This study revealed that the frequency of IR in mammals is far more widespread than previously detected using lower coverage RNA-Seq data (Wang et al. 2008) and affects most multiexonic genes. IR was detected to a variable extent between different cell and tissue types, and in general, a higher proportion of retained introns was found in neural and immune cell types, whereas IR was detected to be less frequent in ESCs and muscle cells. Increased IR in neuronal and immune cells was proposed to facilitate a rapid response to external stimuli, within a time frame shorter than that required for de novo transcription and protein synthesis (Ni et al. 2016; Mauger et al. 2016). Thus, IR may represent a tissue-specific process that serves to restrict the translation of proteins only to cells where they are required while concurrently maintaining transcription from the locus in other tissues. To investigate the physiological roles of IR during the cell maturation program, Braunschweig and colleagues analyzed alternative retained introns using RNA-Seq data from a time series of differentiation of cortical glutamatergic neurons from murine ESCs (Hubbard et al. 2013; Braunschweig et al. 2014). Strikingly, the vast majority (~ 90%) of detected differentially retained introns between embryonic stem cells and mature neurons revealed a progressive increase in retention during differentiation. Consistent with this observation and a global regulatory role for IR in the suppression of gene expression, transcripts with increased IR in mature neurons were expressed at significantly lower steady-state levels than transcripts with increased IR in ESCs. These results suggested that IR accompanies the process of functional tuning of the transcriptomes during murine neuronal differentiation. This is done by reducing the expression of transcripts that are not essential or are less required for the physiology of cells in which they were detected. Elimination of these transcripts was achieved through cytoplasmic NMD (Wong et al. 2013). Such a function of IR in transcriptome tuning remains in agreement with a number of other reports, including maturation of cells of the hematopoietic lineage (Yap et al. 2012; Boutz et al. 2015; Pimentel et al. 2016; Edwards et al. 2016; Llorian et al. 2016; Mauger et al. 2016).

Eukaryotic cells responses to stress-related programs are associated with IR (Shalgi et al. 2014; Boutz et al. 2015; Memon et al. 2016; Pimentel et al. 2016; Edwards et al. 2016; Llorian et al. 2016). Shalgi and colleagues examined genome-wide splicing regulation during heat shock in mouse fibroblasts. They observed widespread retention of introns in transcripts not directly involved in the stress response. In contrast, genes required for the immediate response to heat shock, such as protein folding were unaffected. Newly synthesized RNA from these genes appeared to be mostly cotranscriptionally spliced, whereas IR transcripts were posttranscriptionally spliced. Interestingly, transcripts with retained introns were not exported to the cytosol, but were stably maintained in the nucleus. They potentially serve as a pool of precursors that can be readily spliced and activated for recovery of normal gene expression post stress (Shalgi et al. 2014). Similar control of the level of IR transcripts which is independent of the cytoplasmic NMD pathway and relies on the nuclear RNA surveillance machinery, was shown during neurogenesis (Yap et al. 2012).

## Intron retention in human diseases

IR has also been associated with complex disorders thereby providing disease-specific diagnostic biomarkers. A literature survey identified publications describing the association of IR with neuromuscular-neurodegenerative diseases including Alzheimer’s disease (AD) (Xu et al. 2008), ALS/FTD, DM2 and FECD and DMD (Xiao et al. 2008; Sznajder et al. 2018). IR is also widespread among a range of cancers including myelodysplastic syndromes (MDS) and chronic lymphocytic leukemia (CLL) and is described as a mechanism of tumor suppressor inactivation (Dvinge and Bradley 2015; Jung et al. 2015; Memon et al. 2016; Jeromin and Bowser 2017). In recent years, therapeutic strategies aimed at splicing regulation have been developed and are currently being tested in clinical trials for a range of diseases including muscular dystrophy and motor neuron diseases (Scotti and Swanson 2016; Zakharova 2021). It is therefore increasingly important to understand the relationship between IR and diseases. Below, we briefly review some of the studies that linked IR with human disorders.

A work by Sznajder and colleagues identified specific features of retained introns that contribute to their misprocessing (Sznajder et al. 2018). The authors took advantage of the presence of elongated microsatellites in introns of the genes linked with some of the human hereditary diseases. These included DM2 (CCTG^exp^, repeat expansion), ALS/FTD (GGGGCC^exp^), FECD (CTG^exp^), spinocerebellar ataxias type 10 (ATTCT^exp^), type 37 (ATTTC^exp^), and Friedreich’s ataxia (GAA^exp^) (Wojciechowska and Krzyzosiak 2011). Based on their results, the stable RNA secondary structure adopted by the expanded and highly polymorphic repeats in mutant transcripts has an effect on host intron splicing (Mirkin 2007; Zhang and Ashizawa 2017). In particular, highly structured GC-rich expansions (CTG, CCTG and GGGGCC), disrupted splicing of their host introns when studied in various human tissues and cultured cells from patients. Such misprocessing of mutant introns was not detected for A/AU-rich microsatellites of weak RNA structures (Sznajder et al. 2018). Interestingly, in FECD, DM2 and ALS/FTD disorders, aberrant processing of mutation-harboring intron RNAs occurs along with dysregulation of developmental programs of alternative splicing (Ranum and Day 2004; Cooper et al. 2009). This suggests that GC-rich intronic microsatellite expansions, by altering RNA structure and/or splicing factor accessibility, impair spliceosome recruitment. This in turn could cause misprocessing of host introns in affected genes and trigger pathogenesis. IR has also been implicated in AD. As shown by Xu and coworkers, neuronal expression of apolipoprotein E4 (APOE4) isoform associated with AD pathogenesis, is controlled by the presence or absence of intron 3 in *APOE4* (Xu et al. 2008). In the primary neuron transfection experiment the authors found that expression of the APOE4 isoform was significantly higher when intron 3 was included in their cDNA construct, while lower when it was excluded. Since the APOE4 isoform has been found to increase Alzheimer’s risk (Sienski et al. 2021), this result suggests an association between IR and AD. In ALS transgenic mice degeneration of motor neurons has been linked to overexpression of the peripherin (PRPH) and associated with IR events. Xiao and coworkers have identified a novel splicing variant of the *PRPH* retaining introns 3 and 4 (Xiao et al. 2008). The IR-containing mRNA of peripherin was found to be expressed at a low stoichiometric level in ALS mouse motor neurons. When its expression is upregulated, it leads to the aggregation of peripherin, suggesting that the abnormal splicing retaining introns 3 and 4 produces a splice isoform that in ALS is prone to aggregation.

Several studies have identified an association between IR and cancers. Zhang and coauthors used whole transcriptome sequencing data from five lung adenocarcinoma tissues and matched normal tissues to detect IR (Zhang et al. 2014). A large number of IR events were found in both tissue types, i.e., 2,340 and 1,422 genes contained only tumor-specific and normal tissue-specific retention events, respectively. Subsequent functional analysis indicated that genes with tumor-specific retention include known lung cancer driver genes, e.g., EGFR, ROS1, and RUNX1, and are enriched in pathways that are important in carcinogenesis. IR in these genes causes frameshift, which generally invokes NMD and reduces the expression of mRNAs. These over-expressed or highly mutable driver genes may have a protective effect in patients. In another study by Jung et al. the authors demonstrated that IR is a mechanism leading to the inactivation of tumor suppressor genes (Jung et al. 2015). By analyzing the RNA-seq and exome data from approximately 2000 cancer patients, they determined that at least 163 of the 900 splice-disrupted somatic exon single-nucleotide variants caused IR in an allele-specific manner and were enriched in tumor suppressor genes. Another study of the association between IR and cancers was conducted by Dvinge and Bradley who analyzed the genome-wide RNA splicing patterns of 805 matched tumor and control samples from 16 cancers (Dvinge and Bradley 2015). They found that in cancers, abnormal RNA splicing occurs in the form of IR. They also used the transcriptomic data generated by the Cancer Genome Atlas project to identify large-scale differences in RNA splicing between the cancer and control samples. In all the cancers except breast cancer, IR events were upregulated in comparison to controls. This finding suggests that IR is a common factor associated with tumorigenesis. Finally, through genome-wide quantitative analysis and unsupervised clustering analysis, Dvinge and Bradley confirmed that although some retained introns are shared by the majority of cancer types, most are either present at a low frequency in multiple cancers or unique to primary cancers. Clustering results showed that cancers originating from similar tissues, such as the colon and rectum, have similar patterns of IR.

## Recursive splicing of long introns

**Fig. 4:**
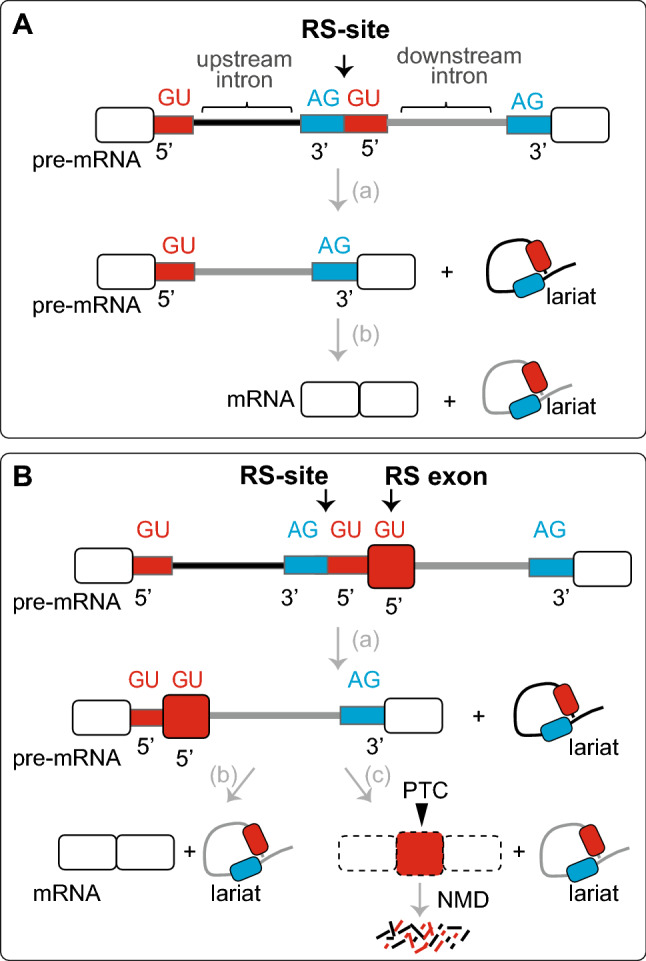
Recursive splicing (RS) of large introns. **A** In *Drosophila*, RS occurs via intronic cryptic sites called recursive splice sites (RS-sites). Each RS-site contains an AGGU sequence motif comprising a pair of juxtaposed 3′ and 5′ splice sites. These splice sites divide a long intron into two or more segments to be sequentially spliced out. This is similar to the signals normally found at the beginning and end of an intron. The RS-site initially functions as a 3′ splice site and pairs with the upstream 5′ splice site to remove the upstream intronic segment (**a**). The reconstituted 5′ splice site interacts with the downstream 3′ splice site, allowing an RS-site to function sequentially as an acceptor and then a donor for splicing of the intronic segment(s) (**b**). As the process continues from one RS-site to the next, small loops of lariat RNA are released from the intron, eventually joining two distant exons. **B** In vertebrates, the RS process involves exon-like events and requires an RS-exon downstream of the RS-site. The site matches the AGGU motif, i.e., the 3′ splice site consensus (AG) immediately followed by the 5′ splice site consensus (GU). The 3′ splice sites of RS-sites need to be sufficiently strong to be recognized by the spliceosome at the first step of recursive splicing (**a**) to be spliced out of the final transcripts (**b**). In some isoforms, the RS exon contains a PTC, and its inclusion decreases mRNA stability via NMD (**c**). As the RS process continues, small loops of lariats are released from the intron

Conventional splicing of a pre-RNA involves the removal of introns as single units in a two-step catalytic reaction. However, processing some large introns > 10 k nt becomes challenging as they are excised from their maturing transcripts by splicing of multiple consecutive subfragments in a process known as recursive splicing. The RS process was first identified in the 73 k nt long intron of the *Ultrabithorax (Ubx)* gene of *Drosophila* (Hatton et al. 1998), and was subsequently experimentally validated in three other genes of the fruit fly i.e., *Kuzbanian*, *Outspread,* and *Frizzled* (Burnette et al. 2005; Conklin et al. 2005). In *Drosophila*, RS occurs via intronic cryptic splice sites, called recursive splice sites (RS-sites) or ratchet points (RPs). Each RS-site contains an AGGU sequence motif comprising a pair of juxtaposed 3′ and 5′ splice sites (Fig. 4). This is similar to the signals normally found at the end and the beginning of an intron, which divides a long intron into two or more segments to be sequentially spliced out. The RS-site initially functions as a 3′ splice site, paired with the upstream 5′ splice site to remove the upstream intronic segment. The reconstituted 5′ splice site then interacts with the downstream 3′ splice site, allowing an RS-site to function sequentially as an acceptor and then a donor for splicing of the intronic segment(s). As the process continues from one RS-site to the next, small loops of lariat RNA are released from the intron eventually joining two distant exons. Unlike canonical splicing, RS events leave no blueprint of the final mRNA, and the only direct evidence of RS is the splicing intermediates. However, the unstable nature of these intermediates and difficulty in capturing them has led to them being overlooked for a long time. Nevertheless, recent advances in high-throughput RNA-Seq have substantially increased the ability to detect RS events. Earlier RS analysis by Duff and coworkers using poly(A) + -selected RNA-seq data (~ 10 billion RNA-seq reads) which derive predominantly from mature transcripts identified 130 recursively spliced introns in flies (Duff et al. 2015). The number of RS-sites per intron ranged from one to six. For example, in the *luna* gene containing a 108 kb intron, five RPs were found, and the intron was removed in a six-step RS. Although, the RS-sites were enriched in large introns over 24 k nt, not all large introns contained these sites and intronic segments removed by RS ranged from ~ 2 k nt to over 60 k nt (Duff et al. 2015). Using this catalog of recursive sites, Duff and coworkers confirmed that recursive splicing is a conserved mechanism to excise constitutive introns, requires canonical splicing machinery, and only occurs in the longest 3% of *Drosophila* introns. More comprehensive analysis of RS in *Drosophila* was later reported from the Burge laboratory (Pai et al. 2018). Using new computational approaches and nascent RNA sequencing data highly enriched for splicing intermediates, Pai and coworkers discovered greater numbers of RS sites (2–4 times more), using less than 1/20th as many reads as used by Duff et al. (2015), supporting the potential of nascent RNA analysis for identification of recursive splice sites. Their analysis determined that very many long introns (> 40 k nt in length) have recursive sites, with the majority (~ 70%) of such introns containing at least one recursive site, and the number of these sites increased roughly linearly with intron length. Overall, they detected 539 candidate recursive sites in 379 fly introns and 98 introns contained multiple recursive sites, with up to seven sites observed in a single intron e.g., intron 1 of the tenascin major (*Ten-m*) gene contains five recursive sites, two of which were previously unknown. Recursively spliced introns were enriched in first introns, which are longer than non-first introns, relative to subsequent introns in fly genes (Pai et al. 2018). Altogether, these results suggests that recursive splicing is the prevalent mechanism by which very large fly introns are excised, however; its function and components remain elusive, and it remains unknown why some long introns are recursively spliced while others are not.

Despite the fact that approximately half of human protein-coding genes have introns over 24 k nt long and contain motifs similar to *Drosophila* RS-sites, only a handful of recursively spliced introns was initially identified in humans, mostly in genes involved in brain development, despite the greater abundance of long introns in vertebrate genomes (Duff et al. 2015; Sibley et al. 2015). Duff et al. analyzed total RNA-Seq datasets from twenty human tissues and identified five RS-sites in four genes, *HS6ST3, CADM2, ROBO2, and PDE4D* (Duff et al. 2015). In an independent study by Sibley et al. global RNA analysis of total RNA-Seq from human postmortem brains identified highly conserved RS-sites in four other genes i.e., *ANK3, CADM1, OPCML,* and *NCAM1.* Introns harboring these RS-sites were among the longest across vertebrates (Sibley et al. 2015). Interestingly, both studies demonstrated that RS events in humans utilize different splicing mechanism compared to those described in *Drosophila*. While no nucleotide RS exons are found in the fruit fly, in vertebrates, the RS process involves exon-like events, such as initial exon definition, and requires an RS-exon downstream of the AGGU RS-site (Fig. 4) (Sibley et al. 2015; Joseph et al. 2018; Pai et al. 2018; Zhang et al. 2018). A comprehensive analysis of the genomic features of human RS-sites was performed by Zhang and colleagues, who found that (i) RS-sites are enriched only in long introns, and RS is a mechanism that aids in the removal of such introns; (ii) RS-sites match the AGGU motif, i.e., the 3′ splice site consensus (AG) immediately followed by the 5′ splice site consensus (GU); (iii) the 3′ splice sites of RS-sites need to be sufficiently strong to be recognized by the spliceosome at the first step of recursive splicing and most RS exons have much lower percent-spliced-in (PSI) values than cassette exons and, in most cases, are spliced out of the final transcripts; (iv) the low efficiency of RS-exon inclusion is correlated with differences in GC% content compared to constitutive exons, and while the later tend to have higher GC% content than their long flanking introns, the former exons exhibit the same GC% content; and (v) some isoforms of RS exons may contain PTC, their inclusion suggests that the latter exons have higher GC stability, which is another factor contributing to the low abundance of RS exons (Zhang et al. 2018) (Fig. 4). Most recent study of RS by Wan and coworkers, who established a high-throughput system for labeling and imaging intronic RNA, and directly measured RNA Pol II and the spliceosome working in concert on endogenous human genes, revealed that most introns are removed from pre-mRNAs in smaller pieces rather than spliced as whole units in one step reaction (Wan et al. 2021). Their results have led to a model of stochastic splice site selection based on which unannotated splicing sites (internal RS sites) within introns are used frequently but randomly by the spliceosomes to make many cuts, instead of a single cut, to progressively remove an intron. This process results in the generation of transient splicing intermediates that are source of a final mRNA. Thus, this study by Wan et al., introduced a stochastic view for splicing that has parallels to the prevailing stochastic view of transcription.

Little is known about the RS machinery that regulates the processing of transcription units with long introns in human genes. Because RS is one of the splicing categories, similar to what was described in canonical splicing, it was proposed that RS is cotranscriptionally regulated and thus kinetically coupled with transcriptional elongation by RNA Pol II (Fong and Zhou 2001; de la Mata et al. 2010; Dujardin et al. 2013; Bentley 2014; Saldi et al. 2016). The Pol II elongation rate has been shown to exert an impact on the efficiency of recursive splicing of the fruit fly *Ubx* gene, correlation that has also been investigated in humans (de la Mata et al. 2003; Zhang et al. 2018). The most comprehensive analysis of the process underlining RS-site processing in the human transcriptome was conducted by Zhang and colleagues, who analyzed RNA-Seq data of time-course 4sUDRB (4-thiouridine tagging of nascent RNAs upon synchronization of Pol II elongation with DRB inhibition) from various human cell lines (PA1ovarian carcinoma cells, H9 embryonic stem cells, and forebrain (FB) neuronal progenitor cells differentiated from H9 cells) (Zhang et al. 2018). The authors determine that the RS introns were removed by both recursive splicing and canonical splicing. They detected intronic reads across the predicted branch point in a lariat configuration (full-length or recursive introns), and reads corresponded to canonical splicing spanning the entire length of the intron, including the RS site. These results suggest that recursive splicing in humans, unlike in flies, is not mutually exclusive with canonical splicing and supplements it rather than replacing it. The choice of which splicing mechanism was used appeared to be cell-type specific indicating that RS is part of the cell’s arsenal for long intron removal. Additionally, Zhang and coauthors defined that most RS events rather than cotranscriptionally underwent posttranscriptional recursive splicing. Although, the onset of most RS splicing events lags behind the completion of transcription of RS introns, they proceed in a timely fashion thereafter (Zhang et al. 2018). This suggests that RS genes exhibit high RNA Pol II elongation rates. They have some genomic and epigenetic features known to be positively correlated with fast Pol II elongation rates in humans. These genes are longer and contain significantly prolonged first introns compared to non-RS genes, in addition to their higher densities of histone marks e.g., H3K79me2 and H4K20me1 (Veloso et al. 2014).

## Lariat-derived stable circular introns

Intronic sequences are spliced out of the primary RNA transcript as branched circular RNAs (lariats). They represent a large fraction of unstable molecules, as most of them are destroyed within minutes in the nucleus via the DBR1 enzyme. The enzyme hydrolyzes the 2′–5′ phosphodiester bond (branch point) in lariats to give rise to linear molecules promoting their rapid turnover (Ooi et al. 2001). Many exceptions to this canonical pathway have been described, and if the excised intron is not debranched, it can linger on in the nucleus or can also be exported to the cytoplasm, where it remains a stable circular molecule (Zhang et al. 2013; Talhouarne and Gall 2018). The presence of circular RNAs (circRNAs) was discovered in 2012 (Salzman et al. 2012) and since then three categories of molecules have been identified in mammalian cells and tissues as a result of linear pre-mRNA splicing which involves a head-to-tail (back-splicing) linking process in which a 5ʹ splice donor is joined to an upstream 3ʹ splice acceptor (e.g., the end of exon 2 is joined to the beginning of exon 1) (Czubak et al. 2019). These three categories include (i) exonic circRNAs (formed by the circularization of one or more exons through a back-splicing process), (ii) exonic-intronic circRNAs (formed by back spliced exons containing retained intron(s) being a product of alternative splicing), and (iii) intronic circRNAs obtained by pathways other than back splicing (Zhang et al. 2013). The processing of these noncanonical lariat-derived stable circular introns (sciRNAs) was found to be dependent on a consensus RNA motif containing a 7-nt GU-rich element near the 5′ splice site and an 11-nt C-rich motif close to the BP site. Additionally, these sciRNAs originate from introns that use cytosine rather than adenosine at the branch point, and they retain the 2′–5′ bond after 3ʹ-end trimming of the lariat. Since the debranching enzyme DBR1 is known to be relatively inefficient in linearizing C-branched lariats, it is speculated that a delay in the linearization of such introns may contribute to the formation of a stable complex that is more resistant to debranching. Moreover, these molecules escape debranching, partly, through export to the cytoplasm where there is no DBR1 enzyme.

**Fig. 5:**
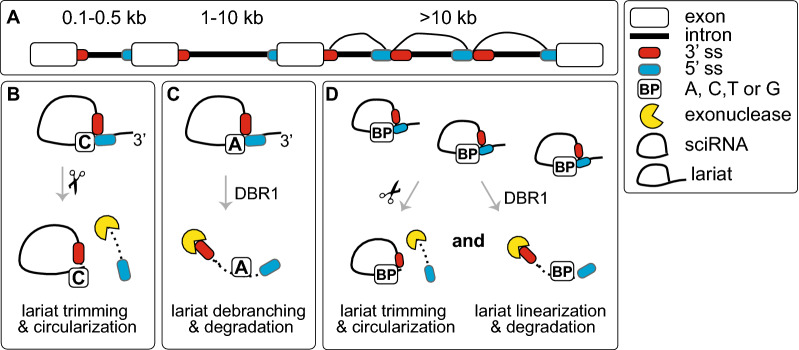
Prospective factors that may influence the fate of excised intronic RNA. **A** Intronic sequences may have different fates post excision from their host pre-mRNAs. **B** When a lariat is derived from an ~ 0.1–0.5 kb long intron having an unusual cytosine (**C**) branch point instead of the canonical adenine (**A**), it may become a stable circular RNA (sciRNA) and may exist either in the nucleus or in the cytoplasm. These sciRNAs might be generated through a failure of intron lariat debranching, which is hypothesized to be incapable of hydrolyzing the 2′–5′ bond when the branch point nucleotide is not an adenine (Jacquier and Rosbash 1986). The formation and processing of sciRNAs depends on a consensus motif containing a 7 nt GU-rich element near the 5′ splice site and an 11 nt C-rich element close to the branch point site. **C** When a medium sized intron of ~ 1–10 kb is released from pre-mRNA, its circularization by branching the 5′ end close to the 3′ end of the intron may be achieved via the canonical adenosine (**A**) branch point. The intron lariat is usually attacked by the DBR1 debranching enzyme and by exonucleases. Thus, the lariat is only an intermediate molecule that is typically rapidly degraded. **D** When large introns over 10 kb are recursively spliced, the resulting fragments in lariat configurations may be either DBR1 linearized in the nucleus and degraded or, following the 3ʹ-end trimming of the lariat tail, they may become stable circular RNAs. The sequence of these lariat-derived sciRNAs corresponds to a lariat without a tail, but includes the 2′–5′ bond. It remains unclear whether the composition of the branch point (BP) guides the fate of excised RS introns, i.e., whether the BP is composed of A, C, G or T

SciRNAs from a variety of genes have been found in the cytoplasm and nucleus of *Xenopus* oocytes, in embryos of *Drosophila*, and in cells and tissues from humans, mice, chickens and fish (Gardner et al. 2012; Talhouarne and Gall 2014, 2018). Molecular analysis of these molecules showed that they were resistant to an α-amanitin inhibitor and were retained in the exposed cells several hours posttreatment, while other conventionally processed introns were degraded. Importantly, these sciRNAs from human, mouse, and chicken cells were usually derived from a single short (~ 100–500 nt) intron per gene (Fig. 5). The biological significance of these circular introns remains obscure; however, it is possible that their cytoplasmic fraction plays a role in the regulation of functional levels of RNA-binding proteins (RBPs) in normal cells and tissues, as proposed for dicer, TDP-43, and snRNPs (Armakola et al. 2012; Li et al. 2016b; Han et al. 2017). However, these molecules may also participate in some disease programs contributing to abnormal depletion and functional insufficiency of splicing factors and other proteins important in the pathogenesis of some human genetic disorders, such as myotonic dystrophy type 1 (DM1) and DM2 (Wojciechowska and Krzyzosiak 2011). Interestingly, the upregulation of various circular RNAs has recently been detected in tissues and cells from DM1 patients and in mouse model of the disease (Czubak et al. 2019; Voellenkle et al. 2019). Their biological role and prospective pathogenic effect are being investigated (Wojciechowska M, unpublished data).

## Concluding remarks

Only approximately 1.5% of DNA contains protein-coding sequences. The other over 98% consists of nonprotein coding regions (Kapranov et al. 2007; Guttman et al. 2009; ENCODE Project Consortium 2012). Evidence is rapidly accumulating that at least some of the noncoding genome is integral to the function of cells, particularly for the control of gene expression. Intronic sequences that account for over 20% of the human genome provide a source of noncoding RNAs, and their processing and biological functions have been of particular interest in recent years (Zhang et al. 2013). It appears that introns are the key source of information for understanding our biology. Their removal from the host transcript is tightly associated with pre-mRNA splicing, and aberrations in this process may result in them retaining in mature mRNA, which may have broad biological consequences. Increasing numbers of identified RI-mRNAs are now recognized as a result of the higher sensitivity of RNA deep sequencing. The levels of RI-mRNAs have been linked with gene regulation during normal development and differentiation, but they have also been recognized as a source of disorders associated with human diseases. Of particular interest is a group of excised introns that escape canonical processing, and their presence in the form of lariats without a tail has been detected in various organisms. Their biological function, interactions with other cellular molecules, and eventual turnover remain elusive, and more research is needed to evaluate whether these byproducts of splicing are indeed unwanted passengers with no biological purpose or whether they represent the key link for the proper physiological regulation of cellular processes.

## Data Availability

Not applicable.

## References

[CR1] Armakola M, Higgins MJ, Figley MD (2012). Inhibition of RNA lariat debranching enzyme suppresses TDP-43 toxicity in ALS disease models. Nat Genet.

[CR2] Barbosa-Morais NL, Irimia M, Pan Q (2012). The evolutionary landscape of alternative splicing in vertebrate species. Science.

[CR3] Bentley DL (2014). Coupling mRNA processing with transcription in time and space. Nat Rev Genet.

[CR4] Berg MG, Singh LN, Younis I (2012). U1 snRNP determines mRNA length and regulates isoform expression. Cell.

[CR5] Berget SM, Sharp PA (1977) A spliced sequence at the 5’-terminus of adenovirus late mRNA. Brookhaven Symp Biol 74(8):3171–3175. 10.1073/pnas.74.8.3171 754866

[CR6] Bieberstein NI, Carrillo Oesterreich F, Straube K, Neugebauer KM (2012). First exon length controls active chromatin signatures and transcription. Cell Rep.

[CR7] Bonadia LC, de Lima Marson FA, Ribeiro JD (2014). CFTR genotype and clinical outcomes of adult patients carried as cystic fibrosis disease. Gene.

[CR8] Boutz PL, Bhutkar A, Sharp PA (2015). Detained introns are a novel, widespread class of post-transcriptionally spliced introns. Genes Dev.

[CR9] Braunschweig U, Barbosa-Morais NL, Pan Q (2014). Widespread intron retention in mammals functionally tunes transcriptomes. Genome Res.

[CR10] Bray M, Prasad S, Dubay JW (1994). A small element from the Mason-Pfizer monkey virus genome makes human immunodeficiency virus type 1 expression and replication Rev-independent. Proc Natl Acad Sci U S A.

[CR11] Breathnach R, Mandel JL, Chambon P (1977). Ovalbumin gene is split in chicken DNA. Nature.

[CR12] Brett D, Pospisil H, Valcárcel J (2002). Alternative splicing and genome complexity. Nat Genet.

[CR13] Broseus L, Ritchie W (2020). Challenges in detecting and quantifying intron retention from next generation sequencing data. Comput Struct Biotechnol J.

[CR14] Burnette JM, Miyamoto-Sato E, Schaub MA (2005). Subdivision of large introns in Drosophila by recursive splicing at nonexonic elements. Genetics.

[CR15] Cho V, Mei Y, Sanny A (2014). The RNA-binding protein hnRNPLL induces a T cell alternative splicing program delineated by differential intron retention in polyadenylated RNA. Genome Biol.

[CR16] Chow LT, Roberts JM, Lewis JB, Broker TR (1977). A map of cytoplasmic RNA transcripts from lytic adenovirus type 2, determined by electron microscopy of RNA:DNA hybrids. Cell.

[CR17] Conklin JF, Goldman A, Lopez AJ (2005). Stabilization and analysis of intron lariats in vivo. Methods San Diego Calif.

[CR18] Cooper TA, Wan L, Dreyfuss G (2009). RNA and disease. Cell.

[CR19] Czubak K, Taylor K, Piasecka A (2019). Global increase in circular RNA levels in myotonic dystrophy. Front Genet.

[CR20] de la Mata M, Alonso CR, Kadener S (2003). A slow RNA polymerase II affects alternative splicing in vivo. Mol Cell.

[CR21] de la Mata M, Lafaille C, Kornblihtt AR (2010). First come, first served revisited: factors affecting the same alternative splicing event have different effects on the relative rates of intron removal. RNA NYN.

[CR22] Duff MO, Olson S, Wei X (2015). Genome-wide identification of zero nucleotide recursive splicing in Drosophila. Nature.

[CR23] Dujardin G, Lafaille C, Petrillo E (2013). Transcriptional elongation and alternative splicing. Biochim Biophys Acta.

[CR24] Dvinge H, Bradley RK (2015). Widespread intron retention diversifies most cancer transcriptomes. Genome Med.

[CR25] Edwards CR, Ritchie W, Wong JJ-L (2016). A dynamic intron retention program in the mammalian megakaryocyte and erythrocyte lineages. Blood.

[CR26] ENCODE Project Consortium (2012). An integrated encyclopedia of DNA elements in the human genome. Nature.

[CR28] Fischer U, Huber J, Boelens WC (1995). The HIV-1 Rev activation domain is a nuclear export signal that accesses an export pathway used by specific cellular RNAs. Cell.

[CR29] Floor SN, Doudna JA (2016). Tunable protein synthesis by transcript isoforms in human cells. Elife.

[CR30] Fong YW, Zhou Q (2001). Stimulatory effect of splicing factors on transcriptional elongation. Nature.

[CR31] Fornerod M, Ohno M, Yoshida M, Mattaj IW (1997). CRM1 is an export receptor for leucine-rich nuclear export signals. Cell.

[CR32] Gaffney DJ, Keightley PD (2004). Unexpected conserved non-coding DNA blocks in mammals. Trends Genet TIG.

[CR33] Gao K, Masuda A, Matsuura T, Ohno K (2008). Human branch point consensus sequence is yUnAy. Nucleic Acids Res.

[CR34] Gardner EJ, Nizami ZF, Talbot CC, Gall JG (2012). Stable intronic sequence RNA (sisRNA), a new class of noncoding RNA from the oocyte nucleus of Xenopus tropicalis. Genes Dev.

[CR35] Ge Y, Porse BT (2014). The functional consequences of intron retention: alternative splicing coupled to NMD as a regulator of gene expression. BioEssays News Rev Mol Cell Dev Biol.

[CR36] Gooding C, Clark F, Wollerton MC (2006). A class of human exons with predicted distant branch points revealed by analysis of AG dinucleotide exclusion zones. Genome Biol.

[CR37] Grüter P, Tabernero C, von Kobbe C (1998). TAP, the human homolog of Mex67p, mediates CTE-dependent RNA export from the nucleus. Mol Cell.

[CR38] Guttman M, Amit I, Garber M (2009). Chromatin signature reveals over a thousand highly conserved large non-coding RNAs in mammals. Nature.

[CR39] Hadzopoulou-Cladaras M, Felber BK, Cladaras C (1989). The rev (trs/art) protein of human immunodeficiency virus type 1 affects viral mRNA and protein expression via a cis-acting sequence in the env region. J Virol.

[CR40] Hammarskjöld ML, Heimer J, Hammarskjöld B (1989). Regulation of human immunodeficiency virus env expression by the rev gene product. J Virol.

[CR41] Han B, Park HK, Ching T (2017). Human DBR1 modulates the recycling of snRNPs to affect alternative RNA splicing and contributes to the suppression of cancer development. Oncogene.

[CR42] Hatton AR, Subramaniam V, Lopez AJ (1998). Generation of alternative ultrabithorax isoforms and stepwise removal of a large intron by resplicing at exon-exon junctions. Mol Cell.

[CR43] Hubbard KS, Gut IM, Lyman ME, McNutt PM (2013). Longitudinal RNA sequencing of the deep transcriptome during neurogenesis of cortical glutamatergic neurons from murine ESCs. F1000Research.

[CR44] Hussein SMI, Puri MC, Tonge PD (2014). Genome-wide characterization of the routes to pluripotency. Nature.

[CR45] Jacob AG, Smith CWJ (2017). Intron retention as a component of regulated gene expression programs. Hum Genet.

[CR46] Jeffreys AJ, Flavell RA (1977). The rabbit beta-globin gene contains a large large insert in the coding sequence. Cell.

[CR47] Jeromin A, Bowser R (2017). Biomarkers in neurodegenerative diseases. Adv Neurobiol.

[CR48] Jones PA (2012). Functions of DNA methylation: islands, start sites, gene bodies and beyond. Nat Rev Genet.

[CR49] Joseph B, Lai EC (2021). The exon junction complex and intron removal prevent re-splicing of mRNA. PLoS Genet.

[CR50] Joseph B, Kondo S, Lai EC (2018). Short cryptic exons mediate recursive splicing in Drosophila. Nat Struct Mol Biol.

[CR51] Jung H, Lee D, Lee J (2015). Intron retention is a widespread mechanism of tumor-suppressor inactivation. Nat Genet.

[CR52] Kalland KH, Szilvay AM, Brokstad KA (1994). The human immunodeficiency virus type 1 Rev protein shuttles between the cytoplasm and nuclear compartments. Mol Cell Biol.

[CR53] Kalsotra A, Cooper TA (2011). Functional consequences of developmentally regulated alternative splicing. Nat Rev Genet.

[CR54] Kapranov P, Cheng J, Dike S (2007). RNA maps reveal new RNA classes and a possible function for pervasive transcription. Science.

[CR55] Lareau LF, Brenner SE (2015). Regulation of splicing factors by alternative splicing and NMD is conserved between kingdoms yet evolutionarily flexible. Mol Biol Evol.

[CR56] Laurent L, Wong E, Li G (2010). Dynamic changes in the human methylome during differentiation. Genome Res.

[CR57] Lewis BP, Green RE, Brenner SE (2003). Evidence for the widespread coupling of alternative splicing and nonsense-mediated mRNA decay in humans. Proc Natl Acad Sci U S A.

[CR58] Li Y, Bor Y-C, Misawa Y (2006). An intron with a constitutive transport element is retained in a Tap messenger RNA. Nature.

[CR59] Li Y, Bor Y-C, Fitzgerald MP (2016). An NXF1 mRNA with a retained intron is expressed in hippocampal and neocortical neurons and is translated into a protein that functions as an Nxf1 cofactor. Mol Biol Cell.

[CR60] Li Z, Wang S, Cheng J (2016). Intron lariat RNA inhibits microRNA biogenesis by sequestering the dicing complex in arabidopsis. PLoS Genet.

[CR61] Lin S, Coutinho-Mansfield G, Wang D (2008). The splicing factor SC35 has an active role in transcriptional elongation. Nat Struct Mol Biol.

[CR62] Llorian M, Gooding C, Bellora N (2016). The alternative splicing program of differentiated smooth muscle cells involves concerted non-productive splicing of post-transcriptional regulators. Nucleic Acids Res.

[CR63] Malim MH, Hauber J, Le SY (1989). The HIV-1 rev trans-activator acts through a structured target sequence to activate nuclear export of unspliced viral mRNA. Nature.

[CR64] Mandel JL, Breathnach R, Gerlinger P (1978). Organization of coding and intervening sequences in the chicken ovalbumin split gene. Cell.

[CR65] Marquez Y, Brown JWS, Simpson C (2012). Transcriptome survey reveals increased complexity of the alternative splicing landscape in Arabidopsis. Genome Res.

[CR66] Marquez Y, Höpfler M, Ayatollahi Z (2015). Unmasking alternative splicing inside protein-coding exons defines exitrons and their role in proteome plasticity. Genome Res.

[CR67] Mauger O, Lemoine F, Scheiffele P (2016). Targeted intron retention and excision for rapid gene regulation in response to neuronal activity. Neuron.

[CR68] Maunakea AK, Chepelev I, Cui K, Zhao K (2013). Intragenic DNA methylation modulates alternative splicing by recruiting MeCP2 to promote exon recognition. Cell Res.

[CR69] McGlincy NJ, Smith CWJ (2008). Alternative splicing resulting in nonsense-mediated mRNA decay: what is the meaning of nonsense?. Trends Biochem Sci.

[CR70] Memon D, Dawson K, Smowton CS (2016). Hypoxia-driven splicing into noncoding isoforms regulates the DNA damage response. NPJ Genomic Med.

[CR71] Mercer TR, Clark MB, Andersen SB (2015). Genome-wide discovery of human splicing branchpoints. Genome Res.

[CR72] Merkin J, Russell C, Chen P, Burge CB (2012). Evolutionary dynamics of gene and isoform regulation in Mammalian tissues. Science.

[CR73] Meyer BE, Malim MH (1994). The HIV-1 Rev trans-activator shuttles between the nucleus and the cytoplasm. Genes Dev.

[CR74] Middleton R, Gao D, Thomas A (2017). IRFinder: assessing the impact of intron retention on mammalian gene expression. Genome Biol.

[CR75] Mirkin SM (2007). Expandable DNA repeats and human disease. Nature.

[CR76] Monteuuis G, Wong JJL, Bailey CG (2019). The changing paradigm of intron retention: regulation, ramifications and recipes. Nucleic Acids Res.

[CR77] Munding EM, Shiue L, Katzman S (2013). Competition between pre-mRNAs for the splicing machinery drives global regulation of splicing. Mol Cell.

[CR78] Ner-Gaon H, Halachmi R, Savaldi-Goldstein S (2004). Intron retention is a major phenomenon in alternative splicing in Arabidopsis. Plant J Cell Mol Biol.

[CR79] Ni T, Yang W, Han M (2016). Global intron retention mediated gene regulation during CD4+ T cell activation. Nucleic Acids Res.

[CR80] Nilsen TW, Graveley BR (2010). Expansion of the eukaryotic proteome by alternative splicing. Nature.

[CR81] Ninomiya K, Kataoka N, Hagiwara M (2011). Stress-responsive maturation of Clk1/4 pre-mRNAs promotes phosphorylation of SR splicing factor. J Cell Biol.

[CR82] Nixon JEJ, Wang A, Morrison HG (2002). A spliceosomal intron in Giardia lamblia. Proc Natl Acad Sci USA.

[CR83] Ooi SL, Dann C, Nam K (2001). RNA lariat debranching enzyme. Methods Enzymol.

[CR84] Pai AA, Paggi JM, Yan P (2018). Numerous recursive sites contribute to accuracy of splicing in long introns in flies. PLoS Genet.

[CR85] Pimentel H, Parra M, Gee SL (2016). A dynamic intron retention program enriched in RNA processing genes regulates gene expression during terminal erythropoiesis. Nucleic Acids Res.

[CR86] Ranum LPW, Day JW (2004). Pathogenic RNA repeats: an expanding role in genetic disease. Trends Genet TIG.

[CR87] Rekosh D, Hammarskjold M-L (2018). Intron retention in viruses and cellular genes: detention, border controls and passports. Wiley Interdiscip Rev RNA.

[CR89] Rogozin IB, Carmel L, Csuros M, Koonin EV (2012). Origin and evolution of spliceosomal introns. Biol Direct.

[CR90] Roy B, Haupt LM, Griffiths LR (2013). Review: alternative splicing (AS) of genes as an approach for generating protein complexity. Curr Genomics.

[CR91] Sakabe NJ, de Souza SJ (2007). Sequence features responsible for intron retention in human. BMC Genomics.

[CR92] Saldi T, Cortazar MA, Sheridan RM, Bentley DL (2016). Coupling of RNA polymerase II transcription elongation with pre-mRNA splicing. J Mol Biol.

[CR93] Saltzman AL, Kim YK, Pan Q (2008). Regulation of multiple core spliceosomal proteins by alternative splicing-coupled nonsense-mediated mRNA decay. Mol Cell Biol.

[CR94] Salzman J, Gawad C, Wang PL (2012). Circular RNAs are the predominant transcript isoform from hundreds of human genes in diverse cell types. PLoS One.

[CR95] Schmitz U, Pinello N, Jia F (2017). Intron retention enhances gene regulatory complexity in vertebrates. Genome Biol.

[CR96] Scotti MM, Swanson MS (2016). RNA mis-splicing in disease. Nat Rev Genet.

[CR97] Shalgi R, Hurt JA, Lindquist S, Burge CB (2014). Widespread inhibition of posttranscriptional splicing shapes the cellular transcriptome following heat shock. Cell Rep.

[CR98] Shukla S, Kavak E, Gregory M (2011). CTCF-promoted RNA polymerase II pausing links DNA methylation to splicing. Nature.

[CR99] Sibley CR, Emmett W, Blazquez L (2015). Recursive splicing in long vertebrate genes. Nature.

[CR100] Sienski G, Narayan P, Bonner JM (2021). APOE4 disrupts intracellular lipid homeostasis in human iPSC-derived glia. Sci Transl Med.

[CR101] Sterne-Weiler T, Martinez-Nunez RT, Howard JM (2013). Frac-seq reveals isoform-specific recruitment to polyribosomes. Genome Res.

[CR102] Sterrantino M, Fuso A, Pierandrei S (2021). Quantitative evaluation of CFTR pre-mRNA splicing dependent on the (TG)mTn poly-variant tract. Diagn Basel Switz.

[CR103] Syed NH, Kalyna M, Marquez Y (2012). Alternative splicing in plants–coming of age. Trends Plant Sci.

[CR104] Sznajder ŁJ, Thomas JD, Carrell EM (2018). Intron retention induced by microsatellite expansions as a disease biomarker. Proc Natl Acad Sci USA.

[CR105] Tahmasebi S, Jafarnejad SM, Tam IS (2016). Control of embryonic stem cell self-renewal and differentiation via coordinated alternative splicing and translation of YY2. Proc Natl Acad Sci USA.

[CR106] Talhouarne GJS, Gall JG (2014). Lariat intronic RNAs in the cytoplasm of Xenopus tropicalis oocytes. RNA NYN.

[CR107] Talhouarne GJS, Gall JG (2018). Lariat intronic RNAs in the cytoplasm of vertebrate cells. Proc Natl Acad Sci USA.

[CR108] Veloso A, Kirkconnell KS, Magnuson B (2014). Rate of elongation by RNA polymerase II is associated with specific gene features and epigenetic modifications. Genome Res.

[CR109] Voellenkle C, Perfetti A, Carrara M (2019). Dysregulation of circular RNAs in myotonic dystrophy type 1. Int J Mol Sci.

[CR110] Wan Y, Anastasakis DG, Rodriguez J (2021). Dynamic imaging of nascent RNA reveals general principles of transcription dynamics and stochastic splice site selection. Cell.

[CR111] Wang ET, Sandberg R, Luo S (2008). Alternative isoform regulation in human tissue transcriptomes. Nature.

[CR112] Wang ET, Ward AJ, Cherone JM (2015). Antagonistic regulation of mRNA expression and splicing by CELF and MBNL proteins. Genome Res.

[CR113] Weatheritt RJ, Sterne-Weiler T, Blencowe BJ (2016). The ribosome-engaged landscape of alternative splicing. Nat Struct Mol Biol.

[CR114] Weischenfeldt J, Waage J, Tian G (2012). Mammalian tissues defective in nonsense-mediated mRNA decay display highly aberrant splicing patterns. Genome Biol.

[CR115] Wojciechowska M, Krzyzosiak WJ (2011). Cellular toxicity of expanded RNA repeats: focus on RNA foci. Hum Mol Genet.

[CR116] Wong JJ-L, Ritchie W, Ebner OA (2013). Orchestrated intron retention regulates normal granulocyte differentiation. Cell.

[CR117] Wong JJ-L, Au AYM, Ritchie W, Rasko JEJ (2016). Intron retention in mRNA: No longer nonsense: known and putative roles of intron retention in normal and disease biology. BioEssays News Rev Mol Cell Dev Biol.

[CR118] Wong JJ-L, Gao D, Nguyen TV (2017). Intron retention is regulated by altered MeCP2-mediated splicing factor recruitment. Nat Commun.

[CR119] Wu J, Xiao J, Wang L (2013). Systematic analysis of intron size and abundance parameters in diverse lineages. Sci China Life Sci.

[CR120] Xiao S, Tjostheim S, Sanelli T (2008). An aggregate-inducing peripherin isoform generated through intron retention is upregulated in amyotrophic lateral sclerosis and associated with disease pathology. J Neurosci off J Soc Neurosci.

[CR121] Xu Q, Walker D, Bernardo A (2008). Intron-3 retention/splicing controls neuronal expression of apolipoprotein E in the CNS. J Neurosci off J Soc Neurosci.

[CR122] Yap K, Lim ZQ, Khandelia P (2012). Coordinated regulation of neuronal mRNA steady-state levels through developmentally controlled intron retention. Genes Dev.

[CR123] Zakharova M (2021). Modern approaches in gene therapy of motor neuron diseases. Med Res Rev.

[CR124] Zhang N, Ashizawa T (2017). RNA toxicity and foci formation in microsatellite expansion diseases. Curr Opin Genet Dev.

[CR125] Zhang Y, Zhang X-O, Chen T (2013). Circular intronic long noncoding RNAs. Mol Cell.

[CR126] Zhang Q, Li H, Jin H (2014). The global landscape of intron retentions in lung adenocarcinoma. BMC Med Genomics.

[CR127] Zhang X-O, Fu Y, Mou H (2018). The temporal landscape of recursive splicing during Pol II transcription elongation in human cells. PLoS Genet.

